# A Rare Case of Lemierre-Like Syndrome: A Case Report and Literature Review

**DOI:** 10.1155/2018/9613493

**Published:** 2018-04-01

**Authors:** Judy Ibrahim, Muhammad Bassel Noureddin, Ali Lootah, Aisha Al Khalidi, Ghassan Ghatasheh, Hossam Al Tatari

**Affiliations:** ^1^Department of Academic Affairs, Tawam Hospital, Al Ain, UAE; ^2^Pediatrics Department, General Pediatrics Division, Tawam Hospital, Al Ain, UAE; ^3^Pediatrics Department, Pediatric Infectious Diseases Division, Tawam Hospital, Al Ain, UAE

## Abstract

Lemierre's syndrome (LS) is a serious rare complication of oropharyngeal infections. It is characterized by thrombosis of internal jugular vein that rapidly progresses into sepsis and is typically caused by anaerobes. Most of the reported cases have been linked to *Fusobacterium necrophorum*; however, there are a handful of reported cases due to aerobes. It is primarily the disease of healthy young adults and can present in school-aged children. The early recognition and treatment of this complication results in resolution of the illness; nevertheless, there have been some concerns about chronic venous insufficiency as a long-term complication. We report a case of a 6-year-old boy, who presented with fever and headache with a history of sore throat. His blood culture was positive for *group A Streptococcus* (GAS) and was subsequently found to have internal jugular vein, sigmoid, and transverse sinus vein thrombosis.

## 1. Introduction

Lemierre's syndrome (LS) is a rare but fatal complication of oropharyngeal infections. It is characterized by thrombosis of internal jugular vein that rapidly progresses into sepsis and is typically caused by the anaerobe *Fusobacterium necrophorum*. However, there are reported cases of Lemierre-like syndrome (LLS) due to aerobes. It is present in school-aged children and healthy young adults. Timely recognition and prompt treatment are corner stones in prevention of complications.

## 2. Case Presentation

A 6-year-old previously healthy boy presented to our pediatric emergency room complaining of fever and headache for one day. His fever was continuous, reaching a maximum of 39C, and was associated with chills. He had no seizures, no altered level of consciousness, and no lethargy. The headache was mild-to-moderate in intensity, band-like in distribution, improving with analgesia, and associated with nonprojectile vomiting (total of 3 times). His father noted limitation of movement of his neck on the same day. These symptoms were preceded by a two-day prodrome of nasal congestion, minimal cough, and sore throat. Review of systems was otherwise negative.

His past medical history was remarkable for mild intermittent asthma, eczema, and allergic rhinitis. He had adenoidectomy twice (in 2012 and in 2014 with bilateral grommet insertion). He was born at term via normal vaginal delivery after an uneventful pregnancy without any postnatal complications. He had achieved all his developmental milestones and was doing well in grade I at school. His family history was not significant for blood disorders or thrombosis. He was thriving well and was living with his seven healthy siblings in their own house, with their caring highly educated parents.

On examination, the child was febrile but hemodynamically stable. He was well looking, well hydrated, and had negative meningeal signs. His ENT examination was only remarkable for erythematous tonsils. He had no neck findings on the first day of admission; however, on the third day, he started to have right-sided soft-tissue neck swelling. The rest of his examination was unremarkable.

**Figure 1:**
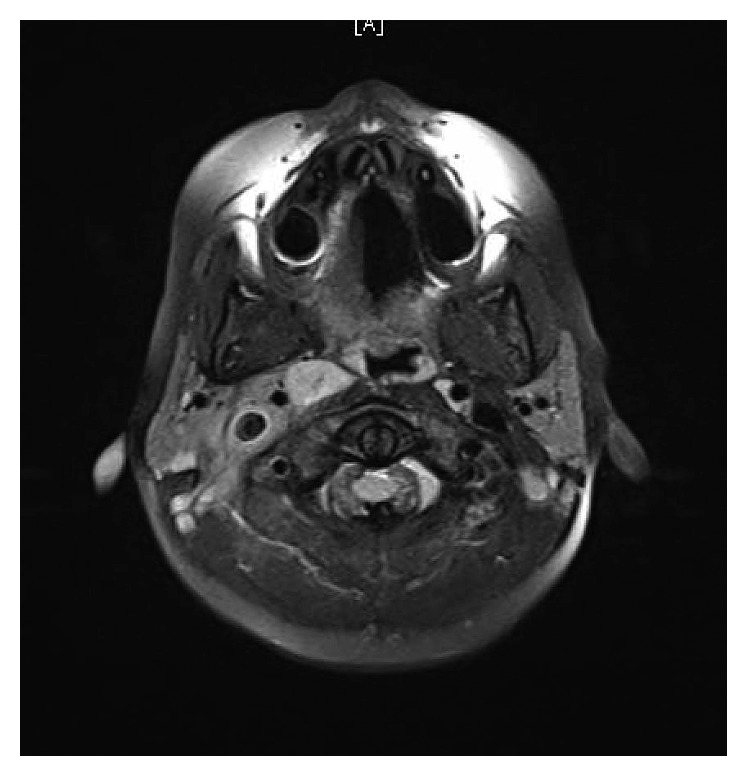
Horizontal section of the MRI of the brain.

**Figure 2:**
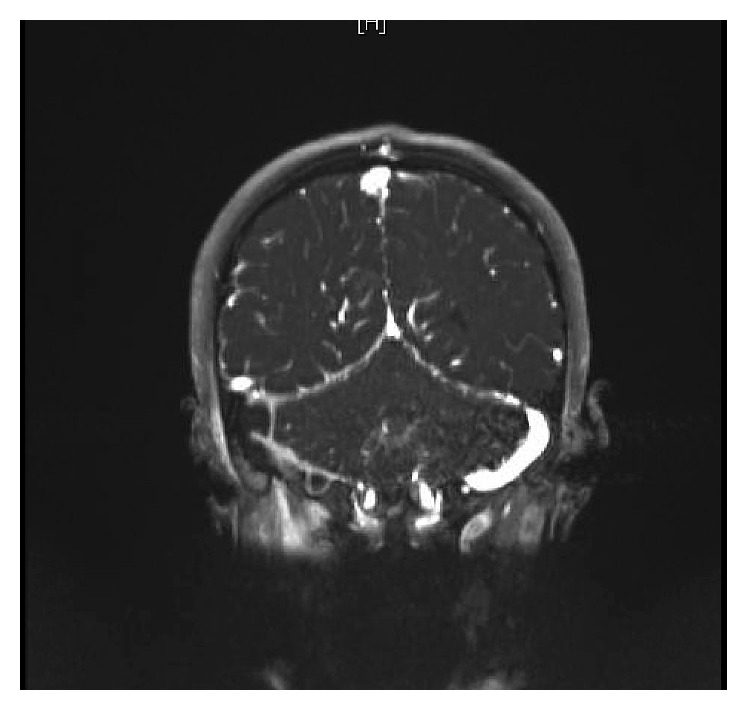
Coronal view of the MRI brain.

**Figure 3:**
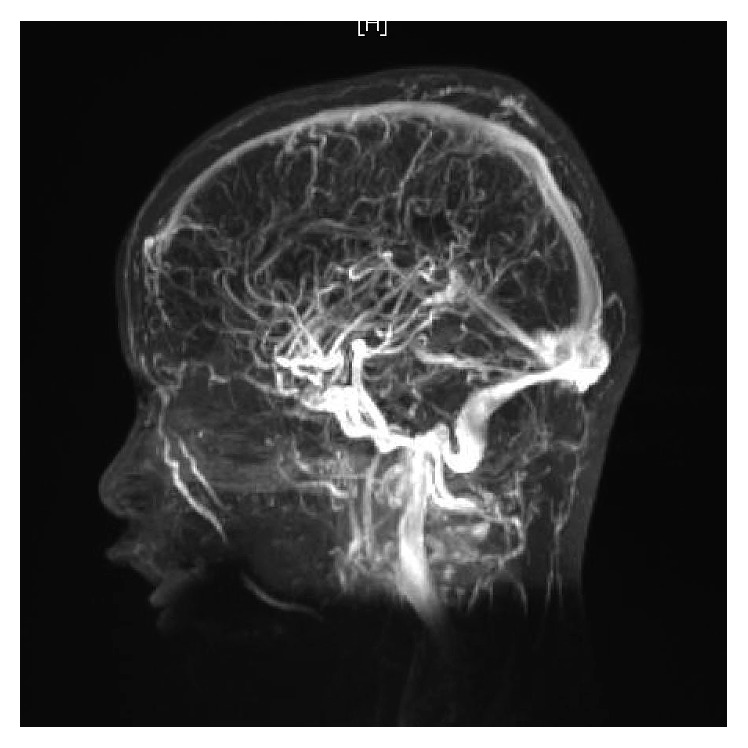
MRV of the brain, showing filling defects in the transverse, sigmoid, and internal jugular veins.

**Figure 4:**
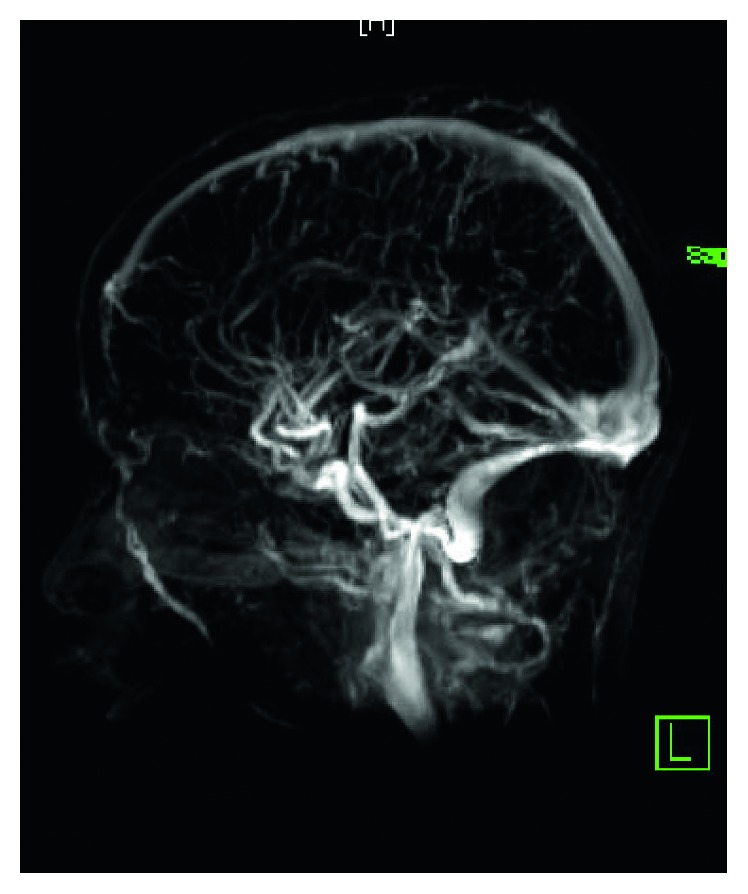
MRV of the brain, showing filling defects in the transverse, sigmoid, and internal jugular veins.

WBC was 18.2 × 10^9^/L, with neutrophilia (14.94 × 10^9^/L). Labs were otherwise unimpressive including urea and electrolytes panel, urine analysis and culture. At 11 hours of admission, blood culture grew Gram-positive cocci in chains, identified later to be *group A Streptococci*. Note that blood cultures are routinely cultured for both aerobes and anaerobes at our institute. LP was tried twice in the emergency room without success. Because of concerns about intracranial involvement, an MRI of the head and neck was obtained. The MRI revealed evidence of venous sinus thrombosis involving the right transverse and sigmoid sinuses as well as the upper part of the jugular vein. The brain parenchymal intensity was normal, and there were no focal lesions (Figures 1 and 2). These findings were confirmed by MRV of the brain (Figures 3 and 4).

The child was initially started on ceftriaxone upon admission. However, the above findings increased the suspicion of Lemierre's syndrome, and therefore metronidazole and enoxaparin were added. Multiple repeated blood cultures (including specific anaerobic ones) were obtained during the admission, all of which were negative. He received a total of six weeks of treatment. A follow-up MRI and MRV of the brain and neck showed significant improvement of the thrombus and evidence of recanalization.

Thrombophilia screening was obtained after completing the enoxaperin therapy and was within normal.

## 3. Discussion

LS, also known as necrobacillosis or postanginal sepsis, is a rare life-threatening complication of oropharyngeal infections. It was first described in the early 1900s by Courmont, Cade, Goodman, Mosher, and Scott muller. In 1936, Andre Lemierre linked the syndrome to an anaerobic sepsis as a consequence of an oropharyngeal infection. He reported 20 cases of anaerobic septicemia, of which 18 died. These cases were mainly, but not exclusively, caused by *Fusobacterium necrophorum* (in up to 71.2% of reported cases [1]).

Later on, a Lemierre-like syndrome (LLS) was described due to *Staphylococcus aureus*, *E. Coli*, and various *Streptococcus* species (peptostreptococci, nonhemolytic streptococci, microaerophilic streptococci, and β-hemolytic streptococci of groups A, B, and C) [2, 3]. EBV has been also reported to be a cause of LLS.

In 2009, Harris et al. reported a 9-year-old boy who developed LLS due to GAS that was isolated in a throat culture, but blood cultures were negative in that case [4]. Before that, in 2005 Wilson and Tierney [5] and in 2007 Anton [6] and Blumberg et al. [7] had separately reported LLS that was solely caused by GAS bacteremia. To our knowledge, our case is the fourth in literature reporting LLS due to GAS bacteremia.

Before the era of antibiotics, the mortality rate of this syndrome was as high as 90%. Recent reported mortality rates are still high averaging around 17% [8]. However, the incidence has been rising in the recent decades, making LS a rising pediatric concern. The fact that cases of LLS due to GAS (which is a relatively common organism in pediatrics) are being reported is a reason for extra concern.

Classically, the focus of the infection is oropharynx (pharyngitis or tonsillitis), but it can follow other head and neck infections such as otitis media and mastoiditis. This is followed by thrombophlebitis of the local veins that ultimately ends by thrombosis of the internal jugular veins. If not treated promptly, this may lead to high-load bacteremia and sepsis, with multiple end-organ failure, lungs being the most commonly involved organ in metastasis.

A constellation of clinical history, examination, microbial isolate, and radiologic findings of thrombosis is used to label the patient with LS or LLS. It is characterized by (1) a history of recent oropharyngeal infection, (2) clinical or radiological evidence of internal jugular vein thrombosis (IJVT), and (3) isolation of a pathogen [3]. A fourth criterion is often added, that is, distant metastatic emboli; frequently pulmonary. CT scan with IV contrast, Doppler of the neck vessels, and MRI (and MRV) are the usual imaging modalities. Timely recognition and prompt start of antibiotics cannot be over emphasized.

The treatment of LS and LLS is drainage of abscesses if any and debridement of any necrotic tissue in parallel to antibiotic therapy. The optimal antibiotic regimen is not well defined. Normally, the abovementioned causative organisms should respond to treatment with penicillin, but due to the high likelihood of resistance by beta-lactamase-producing organisms (such as other *Fusobacterium* species and the concomitant bacteria), the use of penicillin alone may not be sufficient. A combination of high-dose penicillin with metronidazole or monotherapy with clindamycin has been suggested [9]. Like most endovascular infections, the treatment is challenging and often prolonged due to fibrin clot formation that makes it difficult for the antibiotic to penetrate.

Treatment with anticoagulants is controversial. Ridgway et al. recommended the use of anticoagulation in the treatment of LS, to expedite the recovery [10]. However, in a systematic review of reported cases of LS (caused exclusively by *F. necrophorum*), Bondy and Grant concluded that although anticoagulation is commonly used for similar septic emboli, its role in Lemierre's syndrome is unclear [11]. We believe it is advised to use the anticoagulation therapy when the thrombosis propagates retrograde and involves the cavernous sinuses of the brain.

## 4. Conclusion

LS and LLS are rare but very devastating diseases and are on the rise. The fact that such serious infection could be caused by a very common pathogen in childhood such as GAS is concerning. Pediatricians should be very vigilant in recognizing and managing them.
